# Successful re-administration after nano-liposomal irinotecan-induced severe hypersensitivity reaction: a case report

**DOI:** 10.1186/s40780-025-00514-6

**Published:** 2025-12-22

**Authors:** Ryota Kanno, Yoshitaka Saito, Yoh Takekuma, Kazuaki Harada, Yoshito Komatsu, Mitsuru Sugawara

**Affiliations:** 1 Department of Pharmacy, Hokkaido University Hospital, Kita 14-jo, Nishi 5-chome, Kita-ku, Sapporo, 060-8648 Japan; 2 Department of Clinical Pharmaceutics & Therapeutics, Faculty of Pharmaceutical Sciences, Hokkaido University of Science, 4-1, Maeda 7-jo 15-chome, Teine-ku, Sapporo, Hokkaido 006-8585 Japan; 3 Department of Cancer Center, Hokkaido University Hospital, Kita 14-jo, Nishi 5-chome, Kita-ku, Sapporo, 060-8648 Japan; 4 Laboratory of Pharmacokinetics, Faculty of Pharmaceutical Sciences, Hokkaido University, Kita 12-jo, Nishi 6-chome, Kita-ku, Sapporo, 060-0812 Japan

**Keywords:** Hypersensitivity reaction, Nano-liposomal irinotecan, Infusion reaction, Allergy, Complement activation-related pseudo-allergy

## Abstract

**Background:**

Nano-liposomal irinotecan (nal-IRI) combined with 5-fluorouracil (5-FU) and levofolinate has been reported to extend the survival of patients with pancreatic cancer and represents an effective second-line treatment. Nal-IRI encapsulates IRI in liposomes modified with polyethylene glycol (PEGylation), which can lead to infusion reactions. Hypersensitivity reactions (HSR) to chemotherapeutic agents include both allergic and infusion reactions, which are difficult to distinguish. Severe HSR can be fatal; however, discontinuation of the suspected drug directly affects the treatment strategy. We report a case of severe HSR during the first nal-IRI administration, in which treatment was successfully continued with supportive management.

**Case presentation:**

A woman in her early 80s with metastatic pancreatic cancer received nal-IRI + 5-FU + levofolinate as second-line treatment. Five minutes after the initiation of the first nal-IRI infusion, the patient developed a tightening sensation in the head, respiratory distress, convulsions, and loss of consciousness, with percutaneous oxygen saturation dropping to the 80% range. The nal-IRI infusion was immediately discontinued, and emergency management was initiated with intravenous infusion of hydrocortisone, famotidine, d-chlorpheniramine maleate, and oxygen administration, resulting in symptom improvement. Given the limited treatment options for metastatic pancreatic cancer and the patient’s strong desire to continue therapy, we decided to re-administer the regimen with intensified management, increasing dexamethasone to 19.8 mg and adding famotidine 20 mg and d-chlorpheniramine maleate 5 mg. Furthermore, we adopted a three-step dose-escalation method. The infusion was started at one-third of the standard rate, increased to two-thirds after 30 min, and finally adjusted to the full rate after another 30 min, after confirming the absence of HSR symptoms. Consequently, the patient did not exhibit any signs of HSR, and the same administration method was continued for 11 cycles until disease progression.

**Conclusions:**

The novelty of this report lies in presenting, for the first time, the detailed course and process by which treatment was successfully continued in a patient experiencing severe HSR upon initial nal-IRI administration, through enhanced supportive care tailored to the characteristics of the drug and stepwise dose adjustment.

## Background

Pancreatic cancer is the sixth leading cause of cancer-related death worldwide and is associated with a poor prognosis [1]. Nano-liposomal irinotecan (nal-IRI) combined with 5-fluorouracil (5-FU) and levofolinate has been reported to extend overall survival and progression-free survival in patients with metastatic pancreatic ductal adenocarcinoma after prior gemcitabine-based therapy [2]. Nal-IRI encapsulates IRI in polyethylene glycol-modified liposomes (PEGylation), enabling sustained release within the tumor tissue [3]. This PEGylation can induce infusion reactions that are not observed with conventional IRI [4]. Additionally, allergic reactions to IRI itself may occur, and several reports have described such events, although the detailed incidence remains unclear [5, 6]. Hypersensitivity reactions (HSR) to chemotherapeutic agents include both allergic and infusion reactions [7]. Accurately distinguishing allergic from infusion reactions can be challenging in clinical practice. Given that severe HSR can be fatal, early detection and treatment are essential. In general, the suspected drug should be discontinued in cases of severe HSR [8]. However, the potential consequences of treatment discontinuation, such as shortened survival and impaired quality of life due to symptom progression, must also be considered, particularly in pancreatic cancer, where therapeutic options are limited.

We report a case in which a severe HSR developed during the first nal-IRI administration, and treatment was successfully continued using several supportive methods, detailing the treatment process.

## Case presentation

A woman in her early 80s was diagnosed with a pancreatic tail intraductal papillary mucinous neoplasm (IPMN) approximately 16 years ago. She also had a history of right breast cancer and malignant lymphoma, both treated with curative intent and currently in remission. Comorbidities included gallbladder polyps, venous thrombi, hypertension, and type 2 diabetes. The patient was subsequently followed for approximately 9 years with imaging studies. However, the tumor enlarged, and laparoscopic distal pancreatectomy was performed. The histopathological diagnosis was IPMN with high-grade dysplasia. The patient was followed postoperatively for approximately 5 years; however, computed tomography revealed tumor recurrence in the pancreatic body. Based on tumor biopsy results, the patient was diagnosed with invasive intraductal papillary mucinous carcinoma. Two cycles of gemcitabine + S-1 (tegafur/gimeracil/oteracil potassium) were administered preoperatively, followed by residual pancreatic total pancreatectomy; adjuvant S-1 was completed over 6 months. Approximately 2 months after the completion of S-1, metastases were detected in segment seven of the liver and the left lymph node of the celiac artery. The patient was initially treated with gemcitabine + nanoparticle albumin-bound paclitaxel; however, disease progression was confirmed after three cycles of treatment. Therefore, chemotherapy was switched to nal-IRI + 5-FU + levofolinate as second-line treatment, considering the balance between the patient’s age and safety.

Table 1 summarizes the medications used to manage comorbidities at the initiation of nal-IRI. Baseline subjective symptoms included grade 1 peripheral neuropathy in the fingertips and soles, as well as fatigue, attributed to gemcitabine + nanoparticle albumin-bound paclitaxel therapy. The patient had a history of latex allergy (details unknown), but no reported allergies to medications, contrast media, or food.

**Table 1:** Baseline medications

Ingredient name	Dosage	Adaptation
Amlodipine besilate 5.0 mg	Once a day after breakfast	Hypertension
Losartan potassium 50 mg	Once a day after evening	Hypertension
Sitagliptin phosphate hydrate 50 mg	Once a day after breakfast	Type 2 diabetes
Tofogliflozin hydrate 20 mg	Once a day after breakfast	Type 2 diabetes
Vonoprazan fumarate 10 mg	Once a day after breakfast	Gastroduodenal ulcer
Rivaroxaban 15 mg	Once a day after breakfast	Venous thrombus
Pancrelipase 600 mg	Three times a day after every meal	Pancreatic exocrine insufficiency
Rebamipide 100 mg	Three times a day after every meal	Gastroduodenal ulcer
Repaglinide 0.5 mg	Thrice a day between day 1 evening and 2	Type 2 diabetes

Table 2 presents the results of the laboratory tests at the initiation of nal-IRI administration. Inflammatory markers, including white blood cell or eosinophil counts, as well as C-reactive protein levels, were within the normal range. Renal function was normal, although minimal hepatic dysfunction was noted. No significant abnormalities in vital signs were observed on the day of treatment. Accordingly, treatment was initiated considering these conditions.

**Table 2:** Laboratory data on the day of first and second nal-IRI administrations

	First administration	Second administration	Normal range
WBC (/µL)	8300	10,200	3300–8600
Hemoglobin (g/dL)	12.8	13.3	11.6–14.8
Platelet (×10^3^/µL)	628	257	158–348
Neutrophil (/µL)	5320	7487	2000–7500
Lymphocytes (/µL)	1818	1693	1500–4000
Monocytes (/µL)	822	785	200–800
Eosinophil (/µL)	141	92	40–400
Basophil (/µL)	199	143	20–100
Albumin (g/dL)	4.0	4.1	4.1–0.1
T-Bil (mg/dL)	1.0	0.8	0.4–1.5
AST (U/L)	33	29	13–30
ALT (U/L)	24	27	7–23
LD (U/L)	257	267	124–222
γ-GT (U/L)	39	40	9–32
BUN (mg/dL)	13	14	8–20
Cr (mg/dL)	0.6	0.67	0.46–0.79
Ccr (mL/min)	63.4	56.8	-
CRP (mg/dL)	0.11	0.11	0–0.14

nal-IRI, nano-liposomal irinotecan; WBC, white blood cell; T-Bil, total bilirubin; AST, aspartate aminotransferase; ALT, alanine aminotransferase; LD, lactate dehydrogenase; γ-GT, γ-glutamyl transferase; BUN, blood urea nitrogen; Cr, creatinine; Ccr, creatinine clearance; CRP, C-reactive protein

Dexamethasone 9.9 mg and palonosetron 0.75 mg were administered intravenously prior to chemotherapeutic agents as premedication to prevent chemotherapy-induced nausea and vomiting in both metastatic treatments [9]. In addition, levofolinate was administered concurrently with nal-IRI [10]. Because the UDP-glucuronosyltransferase family 1 member A1 (UGT1A1) genotype was unknown, the first dose of nal-IRI was reduced to 70%.

The detailed treatment process is shown in Table 3. Five minutes after the initiation of nal-IRI (approximately 4 mg infusion), the patient experienced a tightening sensation in the head, respiratory distress, and cold sweats. Shortly thereafter, her eyes rolled upward, and tonic convulsions in both upper extremities were observed, with loss of consciousness. These symptoms were diagnosed as an HSR caused by nal-IRI, classified as grade 3 according to the Common Terminology Criteria for Adverse Events v5.0, and administration was immediately discontinued. After discontinuation, consciousness rapidly improved, and the convulsions resolved. However, dyspnea persisted, with percutaneous oxygen saturation (SpO_2_) dropping to the 80% range and systolic blood pressure rising to around 180 mmHg. Therefore, oxygen was immediately administered at a rate of 5 L/min. Simultaneously, rescue medications, comprising 500 mg sodium hydrocortisone succinate, 20 mg famotidine, and 5 mg d-chlorpheniramine maleate dissolved in 50 mL of isotonic sodium chloride solution, were infused intravenously over approximately 5 min. Thereafter, systolic blood pressure decreased gradually and stabilized around 130 mmHg after approximately 30 min, although SpO_2_ remained in the low-to-mid 90% range with 3 L/min of oxygen. The patient was hospitalized for observation. During hospitalization, the patient’s consciousness became clear and vital signs returned to normal; thus, oxygen administration was gradually reduced and discontinued approximately 15 h after treatment initiation, without recurrence of respiratory distress. Vital signs remained normal the following day, and the patient was discharged.

**Table 3:** Timeline of events

Date/Time	Event	Symptoms (CTCAE v5.0)	Treatment/Intervention	Patient’s condition /Outcome
Before the start of secondary treatment	PD	CIPN (grade 1) Fatigue (grade 1)	Planning secondary treatment with nal-IRI + 5-FU + levofolinate	PS1
1st cycle	First nal-IRI administration	None	Premedication: DEX 9.9 mg + palonosetron 0.75 mg (IV) nal-IRI reduced dose (70%)	Start of administration
	5 min after administration	HSR (grade 3)	Administration discontinued immediately	Tightening sensation in the head, respiratory distress, cold sweats, tonic convulsions, and loss of consciousness
			500 mg sodium hydrocortisone succinate, 20 mg famotidine, and 5 mg d-chlorpheniramine maleate were administered (IV) Oxygen administration	Consciousness improved Oxygen administration was initiated because SpO_2_ dropped to the 80% range The patient was hospitalized for observation
	After HSR	None	Observation	As vital signs stabilized, oxygen administration was gradually reduced and discontinued Patient discharged the following day
2nd cycle	Obtain informed consent for re-administration	None	Nal-IRI was increased to the standard dose and administered under hospitalization Premedication (enhanced): DEX 19.8 mg + 20 mg famotidine + 5 mg d- chlorpheniramine maleate Three-step dose-escalation method was implemented	No recurrence of HSR signs or symptoms
3rd-11th cycle	None	None	As in the second cycle, premedication (enhancement) was continued and the infusion rate adjusted	No recurrence of HSR; treatment continued for 7 months (11 cycles total)

PD, progressive disease; CTCAE v5.0, Common Terminology Criteria for Adverse Events v5.0; CIPN, chemotherapy-induced peripheral neuropathy; nal-IRI, nano-liposomal irinotecan; 5-FU, fluorouracil; PS, performance status; DEX, dexamethasone; IV, intravenous; HSR, hyper sensitivity reaction; SpO_2_, percutaneous oxygen saturation

The patient conveyed a strong desire to receive evidence-based chemotherapy, and only a few treatment options were available. Additionally, IRI was suspected as the potential causative agent for HSR in this case; therefore, switching to FOLFIRINOX could carry a similar risk of HSR, along with other intolerable toxicities. Consequently, we considered that continuing treatment with the most effective preventive approach currently available was appropriate in this case. We obtained informed consent with utmost caution from the patient and her family regarding the potential for fatal HSR during subsequent administration, ensuring that both fully understood the associated risks, and it was decided to proceed with treatment during hospitalization. The decision was made to prioritizing early resumption of therapy under enhanced countermeasures, as there was insufficient time to obtain test results, including drug-induced lymphocyte stimulation tests (DLST).

At the time of re-administration, the dose of nal-IRI was increased to the standard dose based on the result of UGT1A1 genotype testing (heterozygous for UGT1A1*6). In addition, the dexamethasone injection dose was increased from 9.9 mg to 19.8 mg, and 20 mg famotidine and 5 mg d-chlorpheniramine maleate were administered intravenously for 30 min prior to nal-IRI, in accordance with anaphylaxis guidelines [11]. Furthermore, the nal-IRI administration rate was adjusted considering the possibility of HSR as an infusion reaction, in accordance with the ESMO clinical practice guidelines [8]. During the first infusion, the rate of nal-IRI administration was approximately 50 mg/h, given that the dose was reduced to 70%. During the second cycle, a three-step increase in the administration rate was adopted. Although this approach is not explicitly defined in the guidelines [8], it was based on established clinical knowledge for managing infusion reactions induced by liposomal formulations [8]. Based on the patient’s body surface area of 1.5 m², 100 mg nal-IRI, dissolved in 500 mL of 5% glucose solution, was administered during the second cycle. Specifically, the infusion was initiated at a rate of approximately 22 mg/h (120 mL/h), one-third of the standard rate. After 30 min, the rate was increased to approximately 44 mg/h (240 mL/h) upon confirming the absence of HSR signs or symptoms. After an additional 30 min, and upon confirming the absence of HSR symptoms, the rate was increased to the standard rate of approximately 66 mg/h (360 mL/h). As a result, the patient did not exhibit any signs of HSR during or after the second administration, and the same administration method was continued thereafter. Grade 1 drowsiness due to d-chlorpheniramine maleate appeared during infusion (approximately 120 min); however, it was transient and deemed not problematic. No other adverse events associated with the countermeasures were observed. In the fifth cycle, we proposed switching to standard administration rates with enhanced premedication, as we hypothesized that this HSR was more consistent with type Ⅰ allergy than with an infusion reaction, based on symptom manifestation. However, the patient declined the proposal because of substantial anxiety. Finally, the patient continued the adjusted administration method for 7 months (11 cycles) until disease progression.

### Discussion and conclusions

To the best of our knowledge, this is the first report to provide a detailed description of the clinical course of severe HSR, a rare adverse event associated with nal-IRI. In a global phase III study on the efficacy of nal-IRI for pancreatic cancer, infusion reactions of any grade occurred in 3.4% of patients, although none exhibited grade 3 or higher symptoms [2]. Palonosetron has been suspected as a potential cause of drug-induced HSR, particularly upon initial administration [12–14]. However, given that palonosetron had been administered five times during prior therapy without symptoms, its likelihood as the causative agent in this case is considered low. Furthermore, baseline concomitant medications had been administered for at least one month, and no drug interactions between these medications and nal-IRI have been confirmed. Therefore, we considered that the symptoms observed in the current case were due to nal-IRI and assessed the relation using the Naranjo Adverse Drug Reaction Probability Scale, with a score of 6, indicating a “Probable” association (Table 4). Furthermore, the score for levofolinate on the Naranjo Drug Reaction Probability Scale was 5, indicating “Probable” association, consistent with the findings for nal-IRI (Table 5). Given that nal-IRI and levofolinate were administered simultaneously in this case, the Naranjo Drug Reaction Probability Scale did not allow for a clear differentiation between the two drugs. However, there appears to be no plausible reason to consider levofolinate as a causative agent. Chemically, it resembles naturally occurring folic acid in the body and is similarly stable [15]. Furthermore, the patient had no history of food allergies. Additionally, a previous report indicated that anaphylaxis did not occur in a total of 6,106 levofolinate doses administered to 494 patients, although there is no post-marketing surveillance of HSR with levofolinate alone [16]. Based on these factors, allergic reactions to levofolinate are considered extremely rare. In contrast, multiple reports implicate nal-IRI as the potential causative agent [4, 17–19]. Consequently, we consider nal-IRI, rather than levofolinate, to be the suspected agent.

**Table 4:** Naranjo adverse reaction probability scale assessment of severe hypersensitivity reaction and nano-liposomal Irinotecan

Question	Yes	No	Do not know	Score
1. Are there previous conclusive reports on this reaction to the drug?	+ 1	0	0	+ 1
2. Did the adverse event appear after the suspected drug was administered?	+ 2	- 1	0	+ 2
3. Did the adverse reaction improve when the drug was discontinued or a specific antagonist was administered?	+ 1	0	0	+ 1
4. Did the adverse reaction reappear when the drug was readministered?	+ 2	- 1	0	- 1
5. Are there alternative causes (other than the drug) that could have, on their own, caused the reaction?	- 1	+ 2	0	+ 2
6. Did the reaction reappear when a placebo was given?	- 1	+ 1	0	0
7. Was the drug detected in blood (or other fluids) in concentrations known to be toxic?	+ 1	0	0	0
8. Was the reaction more severe when the dose was increased, or less severe when the dose was decreased?	+ 1	0	0	0
9. Did the patient have a similar reaction to the same or similar drugs in any previous exposure?	+ 1	0	0	0
10. Was the adverse event confirmed by any objective evidence?	+ 1	0	0	+ 1
Total score				+ 6

A score of 6 indicates a “Probable” association

**Table 5:** Naranjo adverse reaction probability scale assessment of severe hypersensitivity reaction and levofolinate

Question	Yes	No	Do not know	Score
1. Are there previous conclusive reports on this reaction to the drug?	+ 1	0	0	0
2. Did the adverse event appear after the suspected drug was administered?	+ 2	- 1	0	+ 2
3. Did the adverse reaction improve when the drug was discontinued or a specific antagonist was administered?	+ 1	0	0	+ 1
4. Did the adverse reaction reappear when the drug was readministered?	+ 2	- 1	0	- 1
5. Are there alternative causes (other than the drug) that could have, on their own, caused the reaction?	- 1	+ 2	0	+ 2
6. Did the reaction reappear when a placebo was given?	- 1	+ 1	0	0
7. Was the drug detected in blood (or other fluids) in concentrations known to be toxic?	+ 1	0	0	0
8. Was the reaction more severe when the dose was increased, or less severe when the dose was decreased?	+ 1	0	0	0
9. Did the patient have a similar reaction to the same or similar drugs in any previous exposure?	+ 1	0	0	0
10. Was the adverse event confirmed by any objective evidence?	+ 1	0	0	+ 1
Total score				+ 5

A score of 5 indicates a “Probable” association

The pathophysiology of HSR to nal-IRI is thought to involve two primary mechanisms: allergy to the IRI component and infusion reactions attributable to the PEGylation of the liposomal formulation [4–6, 17–21]. Both reactions manifest with immediate symptoms, such as skin manifestations, fever, sweating, and dyspnea within minutes of infusion initiation, making clinical differentiation extremely difficult [4–6, 17–21]. Distinguishing these pathologies directly impacts subsequent treatment strategies. An allergic reaction to IRI is immunoglobulin E (IgE)-dependent, involving the release of mediators from mast cells and basophils upon allergen exposure [5, 6, 21]. Re-administration carries the potential for symptom exacerbation due to repeated exposure [5, 6, 21]. Previous reports indicate that complex desensitization protocols are often necessary to mitigate this risk [5, 6, 21]. If the HSR in this case was considered an allergy, it would be regarded as a typical early-onset type I allergy. DLST is primarily used to diagnose delayed-type IV allergies [22]. Therefore, DLST was considered to have limited utility and was not performed. In contrast, infusion reactions are IgE-independent. For other liposomal formulations, such as pegylated liposomal doxorubicin, symptoms have been reported to improve following repeated exposure (showing tachyphylaxis) and are often manageable by slowing the infusion rate [17–20]. This phenomenon is commonly recognized as complement activation-related pseudo-allergy (CARPA) [17–20]. In the present case, HSR manifested immediately (within 5 min), and both the symptoms and timing of onset corresponded to the previously reported clinical features of HSR associated with irinotecan and liposomal formulations [4–6, 17–21]. Consequently, determining whether to pursue “desensitization” or “rate adjustment” during re-administration was critical in establishing the treatment strategy. In this case, laboratory tests performed before the first nal-IRI administration revealed elevated basophil levels. Basophils are implicated in allergic diseases, and increased basophil counts in peripheral blood have been associated with T-helper 2 cell cytokine-mediated allergic inflammation [23]. Furthermore, basophils are recognized as contributing factors to anaphylaxis [23]. In our patient, the elevated baseline basophil count appeared to predispose her to allergic reactions and anaphylaxis. Consequently, the severe HSR in this case may have been allergic in nature; however, it could not be definitively established. Therefore, we adopted a combined approach in this case. To avoid a complicated desensitization protocol, we strengthened allergy countermeasures with medication and implemented CARPA countermeasures using a three-step dose-escalation method, despite the limited supporting evidence. Specifically, premedication was intensified by increasing the dexamethasone dose to 19.8 mg and adding famotidine 20 mg and d-chlorpheniramine maleate 5 mg [8, 11]. Additionally, the infusion rate was gradually increased in three steps based on a 90-min infusion of 70 mg/m^2^ [8, 11]. These enhanced administration methods successfully prevented HSR recurrence in subsequent cycles. If this case had represented CARPA, which is characterized by tachyphylaxis, it might have been possible to return to the standard administration rate and normal premedication regimen; however, this was not attempted.

If severe HSR occurs, the drug should be discontinued [8]. However, we decided to re-administer nal-IRI based on the following rationale: first, there are few treatment options available for pancreatic cancer [24]; second, although the patient was older, she had a good performance status of 1, and a strong desire to receive treatment. She and her family received adequate information regarding the risk of severe HSR recurrence and understood the risks. Third, several prophylactic reinforcement measures recommended in the guidelines could be implemented under inpatient management [8, 11]. Our report suggests an additional treatment strategy for managing severe HSR in clinical cancer practice, depending on the patient’s circumstances.

In conclusion, this case report demonstrates that safe re-administration of nal-IRI is achievable even after severe HSR during treatment of metastatic pancreatic cancer, a setting with limited therapeutic options. This approach may provide a valuable strategy for managing severe HSR in cancer treatment, where therapeutic strategies are limited.

## Data Availability

All data generated in this study are included in this article. Further inquiries can be directed to the corresponding authors.

## Abbreviation

nal-IRI: Nano-liposomal irinotecan
5-FU: Fluorouracil
PEGylation: Polyethylene glycol-modified
HSR: Hypersensitivity reactions
IPMN: Intraductal papillary mucinous neoplasm
UGT1A1: UDP-glucuronosyltransferase family 1 member A1
SpO2: Percutaneous oxygen saturation
DLST: Drug-induced lymphocyte stimulation tests
IgE: Immunoglobulin E
CARPA: Complement activation-related pseudo-allergy
